# A new predictive model combined of tumor size, lymph nodes count and lymphovascular invasion for survival prognosis in patients with lymph node-negative gastric cancer

**DOI:** 10.18632/oncotarget.11035

**Published:** 2016-08-04

**Authors:** Lin-Yong Zhao, Xiao-Long Chen, Yi-Gao Wang, Yue Xin, Wei-Han Zhang, Yin-Su Wang, Xin-Zu Chen, Kun Yang, Kai Liu, Lian Xu, Bo Zhang, Zhi-Xin Chen, Jia-Ping Chen, Zong-Guang Zhou, Jian-Kun Hu

**Affiliations:** ^1^ Department of Gastrointestinal Surgery, West China Hospital, Sichuan University, Chengdu, China; ^2^ Laboratory of Gastric Cancer, State Key Laboratory of Biotherapy/Collaborative Innovation Center of Biotherapy, West China Hospital, Sichuan University, Chengdu, China; ^3^ West China School of Medicine, Sichuan University, Chengdu, China

**Keywords:** predictive model, gastric cancer, tumor size, lymphovascular invasion, lymph nodes count

## Abstract

**Background:**

Various factors may affect the clinical prognosis of lymph node-negative gastric cancer (GC) patients. This study aimed to provide evaluable prognostic information of combination of tumor size (Ts), lymph nodes count (LNs) and lymphovascular invasion (LVI) in lymph node-negative GC patients.

**Methods:**

A total of 1,019 node-negative GC patients were enrolled in this retrospective study from 2000 to 2010. The cutoff points of Ts and LNs were determined using X-tile and patients were randomly categorized into training and validation sets by the sample size ratio 1:1. The clinicopathologic characteristics were analyzed and survival prognostic factors were identified, whereas the survival prediction accuracy was also compared by C-index during the different independent prognostic factors.

**Results:**

The cutoff points for Ts were 3cm and 5cm, while 14 was the cutoff point for LNs. Age, T stage, Ts, LNs and LVI were identified as independent prognostic factors in node-negative GC patients, and a new prognostic predictive model, TsNL staging system which was composed of Ts, LNs and LVI, was proposed in this study. Compared with T staging system, significant improvement of predictive accuracy for TsNL system was found. Furthermore, nomogram based on TsNL was more accurate in prognostic prediction than that based on Ts, LNs and LVI, separately.

**Conclusions:**

Age, T stage, Ts, LNs and LVI were independent prognostic factors in lymph node-negative GC patients. The TsNL staging system, composed of Ts, LNs and LVI, which was closely associated with clinicopathologic features, may improve the prognostic prediction accuracy in node-negative GC patients.

## INTRODUCTION

Despite declining global incidence, gastric cancer (GC) remains one of the most common malignances nowadays, with the secondary leading cause of cancer-related mortality in China [1]. Being widely regarded to be the most important prognostic indicators for GC, depth of tumor invasion (T stage) and status of lymph nodes (N stage), have been enrolled in tumor-node-metastasis (TNM) staging system not only in the American Joint Committee on Cancer (AJCC) [2] but in the Japanese Gastric Cancer Association (JGCA) [3], which is due to the consideration that, this staging system is able to provide accurate prognostic estimation and guidance of choosing appropriate therapeutic protocols for GC patients, and to distinguish the prognostic differences among several subgroups of patients. Lymph node-negative GC patients have been demonstrated in previous studies [4, 5] to present better survival than those with positive lymph nodes involvement, nevertheless, even among the node-negative patients, the survival rate for certain subgroups were worse than others, and some of them still were at the risk of recurrence or cancer-related death. Although several investigators reported that, apart from the most important prognostic factor, T stage, various clinicopathologic factors such as lymphovascular invasion (LVI) [6–9], tumor size (Ts) [4, 10, 11], lymph node count (LNs) [12–15] and perineural invasion [16], were additionally confirmed as independent prognostic factors which were significantly associated with survival for node-negative GC patients followed curative resection, unfortunately, no consensus on this issue by far has been yet reached and few studies focused on prognostic role of the combination of these prognostic factors [6].

In light of these consideration mentioned above, it is highly necessary to analyze independent prognostic factors among a series of clinicopathologic features for node-negative GC patients underwent curative gastrectomy. Therefore, we conducted this study to identify the independent prognostic factors and to dig out some valuable prognostic information about the combination of these factors, trying to explore a more appropriate staging system based on these identified factors than the well-known prognostic factor, T stage, for precise and accurate prediction of the prognosis on overall survival in node-negative GC patients after curative surgery.

## RESULTS

### Optimal cutoff points for tumor size and lymph nodes count

**Figure 1:**
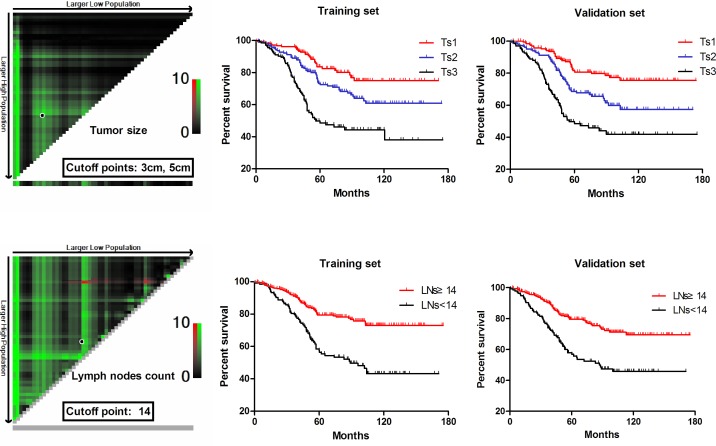
Division of patients by the cutoff points produced by X-tile plot

X-tile plots, constructed in Figure 1, indicated that the optimal cutoff points for tumor size (Ts) were 3.0cm and 5.0cm by minimum P value from log-rank χ^2^ test, based on which patients were divided into three groups, Ts1: ≤ 3cm, Ts2: 3-5cm, Ts3: ≥5cm, with the strongest discriminatory capacity. The count of lymph nodes retrieved in our study ranged from 8 to 59, with a median of 26 and a mean of 25.02 ± 8.80, and according to the optimal cutoff point for the lymph nodes count (LNs), 14, which was produced by X-tile shown in Figure 1, we defined LNs≥14 and LNs < 14 as N0 and N1, respectively. Consequently, a total of 1019 patients enrolled in our study were randomly separated into the training set (*n* = 510) and the validation set (*n* = 509), and there were no significant difference existing between these two sets in terms of different clinicopathologic factors (all of the P* value >0.05, illustrated in Table 1), which meant baseline for the two sets was balanced.

**Table 1:** Correlation between TsNL stage and clinicopathologic factors in the training set and validation set

Factors	Training set							Validation set							P*
	I (n=139)	II (n=129)	III (n=107)	IV (n=99)	V (n=36)	Total (n=510)	P	I (n=131)	II (n=130)	III (n=105)	IV (n=109)	V (n=34)	Total (n=509)	P	
Gender							<0.001							0.003	0.058
Male	94	113	33	88	29	357		101	99	93	71	19	383		
Female	45	16	74	11	7	153		30	31	12	38	15	126		
Age (years)							0.002							0.001	0.095
<65	71	52	39	24	9	185		79	61	32	28	11	211		
≥65	68	77	68	75	27	325		52	69	73	81	23	298		
Tumor location							<0.001							<0.001	0.057
Upper third	21	23	24	15	8	91		22	14	17	11	10	74		
Middle third	40	33	22	41	17	153		27	41	39	79	7	193		
Lower third	78	71	57	36	3	245		81	74	45	13	9	222		
≥2/3 stomach	0	2	4	7	8	21		1	1	4	6	8	20		
Macroscopic type							<0.001							<0.001	0.080
Type 0-II	119	108	81	70	16	394		108	101	73	72	15	369		
Type III-V	20	21	26	29	20	116		23	29	32	37	19	140		
Tumor differentiation							<0.001							<0.001	0.054
Well/Moerately	56	33	16	11	8	124		61	31	25	26	8	151		
Poorly	83	96	91	88	28	386		70	99	80	83	26	358		
Perineural invasion							0.002							0.011	0.234
Negative	120	113	88	73	13	407		112	118	91	89	11	421		
Positive	19	16	19	26	23	103		19	12	14	20	23	88		
T Stage							<0.001							<0.001	0.084
T1-T2	116	88	33	48	5	290		108	82	29	39	4	262		
T3-T4b	23	41	74	51	31	220		23	48	76	70	30	247		
Ts							<0.001							<0.001	0.654
≤3cm (Ts1)	139	33	33	0	0	205		131	32	37	0	0	200		
3-5cm (Ts2)	0	96	45	38	0	179		0	98	50	43	0	191		
≥5cm (Ts3)	0	0	29	61	36	126		0	0	18	66	34	118		
LNs							<0.001							<0.001	0.127
≥14 (N0)	139	102	72	46	0	359		131	107	76	66	0	380		
<14 (N1)	0	27	35	53	36	151		0	23	29	43	34	129		
LVI						<0.001								<0.001	0.259
Negative(L0)	139	117	91	72	0	419		131	112	84	77	0	404		
Positive (L1)	0	12	16	27	36	91		0	18	21	32	34	105		

P*: the difference between the training set and the validation set; Ts: tumor size; LVI: lymphovascular invasion; LNs: lymph nodes count; N0: LNs≥14; N1: LNs<14.

### Multivariate analyses for patients' prognosis and the proposal of TsNL staging system

**Figure 2:**
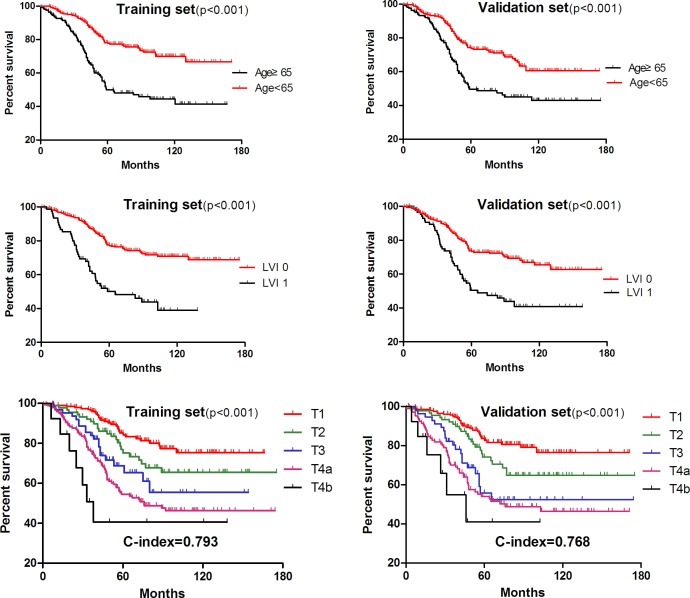
Kaplan-Meier survival analysis in terms of age, LVI and T stage

As demonstrated in Table 2, multivariate analysis by Cox regression model showed that age, tumor size (Ts), lymph nodes count (LNs), lymphovascular invasion (LVI) and T stage were independent prognostic factors of overall survival for lymph node-negative gastric cancer patients both in the training set and validation set. Moreover, survival curves related to these factors were illustrated in Figures 1 & 2, and significant difference was found in terms of all of these independent factors(*p* < 0.001).

**Table 2:** Multivariate analyses of clinicopathologic factors associated with OS by Cox regression model

Factors	Training set (*n* = 510)		Validation set (*n* = 509)	
	HR(95%CI)	*P* value	HR(95%CI)	*P* value
Gender	0.996(0.866-1.105)	0.087	0.896(0.762-1.054)	0.101
Age	1.325(1.101-1.980)	0.039	1.425(1.003-2.027)	0.048
Tumor location	0.894(0.788-1.002)	0.091	0.956(0.897-1.153)	0.132
Macroscopic type	1.003(0.823-1.145)	0.202	1.001(0.891-1.106)	0.156
Tumor differentiation	0.978(0.879-1.084)	0.124	0.961(0.858-1.078)	0.119
Tumor size(Ts)	1.554(1.232-1.967)	0.001	1.442(1.127-1.844)	0.004
Perineural invasion	1.254(0.732-1.629)	0.233	1.132(0.892-1.567)	0.341
Lymph nodes count (LNs)	1.401(1.012-2.275)	0.024	1.698(1.126-2.562)	0.012
Lymphovascular invasion (LVI)	0.612(0.390-0.873)	<0.001	0.536(0.376-0.765)	0.002
T stage	1.439(1.069-1.702)	0.001	1.348(1.178-1.544)	<0.001

OS: overall survival; HR: Hazard Ratio; CI: Confidence Interval.

**Figure 3:**
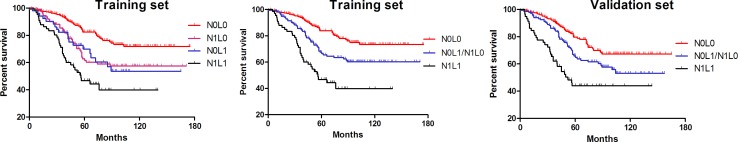
Kaplan-Meier survival analysis in terms of combination of LVI and LNs

**Figure 4:**
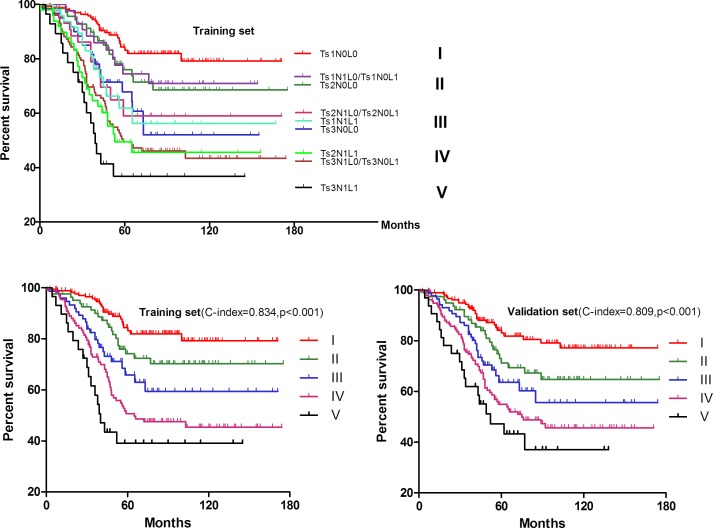
Kaplan-Meier survival analysis of the TsNL staging system

In order to dig out detailed prognostic information of these independent factors, we firstly combined LNs and LVI to make Kaplan-Meier survival analysis and found that there was a cross line between the N0L1 and N1L0 (*p* = 0.498) in Figure 3. In addition, the survival curves suggested a largely improved discriminatory ability after the integration of N0L1 and N1L0 both in the training set (*p* < 0.001) and validation set (*p* < 0.001). Furtherly, Ts and LNs as well as LVI were combined together to make survival analyses in Figure 4, illustrating that overlapping survival curves presented and no significant difference was found between Ts1N1L0/Ts1N0L1 and Ts2N0L0 by log rank test (*p* = 0.732). Interestingly, similarity was also found among Ts1N1L1, Ts3N0L0 and Ts2N1L0/Ts2N0L1 (*p* = 0.429), and between Ts2N1L1 and Ts3N1L0/Ts3N0L1 (*p* = 0.791). Therefore, we tried to integrate them respectively into stage II, III, IV, whereas Ts1N0L0 was regarded as stage I with Ts3N1L1 defined as stage V. Given that this new stage-integrating strategy just mentioned before was surprisingly able to utilize both in the training set and validation set (Figure 4), we proposed a new staging system, TsNL which was composed of Ts, LNs and LVI, illustrated in Table 3.

**Table 3:** TsNL staging system

	N0L0	N0L1/N1L0	N1L1
Ts1	I	II	III
Ts2	II	III	IV
Ts3	III	IV	V

Ts: tumor size; N: lymph nodes count (LNs); L: lymphovascular invasion (LVI);

Ts1:≤3cm; Ts2: 3-5cm; Ts3:≥5cm; N0: LNs≥14; N1: LNs<14; L0: LVI (−); L1: LVI (+).

### Clinicopathologic factors and correlation analysis

Clinicopathologic factors were compared among the five stages, as shown in Table 1. Both in the training set and validation set, TsNL stage was significantly related to gender, age, tumor location, macroscopic type, tumor differentiation and perineural invasion as well as T stage. Compared with the TsNL stage IV and V, patients with stage II and III were found more frequently in male and in the age of ≥65 years, having a higher proportion in macroscopic type 0-II, in well/moderate differentiation and in early T stage as well as negative perineural invasion.

As demonstrated in Table 4, logistic regression analyses were performed respectively to determine the risk factors for those four independent prognostic factors identified by Cox regression analysis. As a result, T stage and Ts were mutually evaluated as the risk factor for each other (*p* < 0.05), indicating that T stage was closely correlated to Ts and that multicollinearity between them was found. That was one of the reason why T stage was not taken into account for our TsNL staging system. However, no correlation was found during other factors, such as Ts, LNs and LVI.

**Table 4:** Logistic regression analysis of the risk factors for the independent prognostic factors

Factors	T stage		Ts			LNs			LVI		
	OR(95%CI)	*p* value		OR(95%CI)	*p* value		OR(95%CI)	*P* value		OR(95%CI)	*p* value
T stage	-	-	2.552(2.314-2.785)		0.009	0.904(0.693-1.421)		0.492	1.105(0.898-1.374)		0.124
Ts	2.478(2.032-2.931)	0.021	-		-	0.929(0.798-1.221)		0.146	0.921(0.793-1.101)		0.145
LNs	0.932(0.643-1.403)	0.402	0.891(0.776-1.164)		0.132	-		-	1.108(0.989-1.385)		0.533
LVI	1.057(0.933-1.439)	0.105	0.903(0.728-1.098)		0.123	1.019(0.913-1.234)		0.365	-		-
Age	1.187(0.982-1.415)	0.063	1.123(0.829-1.428)		0.605	1.172(0.875-1.654)		0.413	1.057(0.933-1.439)		0.105
Gender	1.102(0.994-1.508)	0.201	1.320(0.794-1.508)		0.541	1.026(0.774-1.508)		0.532	1.002(0.849-1.307)		0.243
Tumor location	1.131(0.953-1.457)	0.182	1.036(0.979-1.346)		0.071	1.215(0.789-1.751)		0.323	1.102(0.994-1.508)		0.101
Macroscopic type	1.147(1.089-1.378)	0.041	1.063(0.902-1.278)		0.062	1.009(0.889-1.428)		0.132	1.163(0.872-1.327)		0.198
Perineural invasion	1.016(0.781-1.369)	0.069	1.146(0.891-1.428)		0.091	1.105(0.767-1.323)		0.292	1.263(0.991-1.713)		0.198
Tumor differentiation	1.208(0.665-1.537)	0.219	1.106(0.804-1.425)		0.197	1.326(0.701-1.843)		0.197	1.106(0.934-1.425)		0.067

Ts: tumor size; LNs: lymph nodes count; LVI: lymphovascular invasion; OR: Odds Ratio; CI: Confidence Interval.

### Survival analysis and prognostic accuracy of TsNL staging system

The 1-year, 3-year and 5-year overall survival (OS) rates of each TsNL stage for the training and validation set were shown in Table 5. The 5-year OS of stage I, II, III, IV, V were 86.1%, 75.2%, 66.4%, 48.1%, 40.3% for the training set and 87.8%,72.4%,61.6%,54.1%,43.9% for the validation set, respectively.

**Table 5:** Survival analysis of patients in the training set and validation set in terms of TsNL staging system

	Training set (*n*= 510)						Validation set (*n*= 509)			
Stage	1-yr OS	3-yr OS	5-yr OS	MS (month)		1-yr OS		3-yr OS	5-yr OS	MS(month)
I	94.6%	91.8%	86.1%	107.0(1.8-170.0)		95.1%		90.3%	87.8%	94.3(1.5-173.3)
II	93.4%	88.9%	75.2%	98.2(0.9-172.1)		94.1%		86.6%	72.4%	87.0(1.9-173.1)
III	93.3%	80.5%	66.4%	72.9(2.5-170.8)		93.9%		82.1%	61.6%	68.0(3.2-171.8)
IV	92.5%	72.2%	48.1%	72.8(1.2-172.0)		93.1%		74.6%	54.1%	65.4(1.4-169.0)
V	88.6%	60.4%	40.3%	40.4(0.7-146.0)		89.9%		63.0%	43.9%	45.2(0.9-134.2)

OS: overall survival; MS: median survival time.

**Figure 5:**
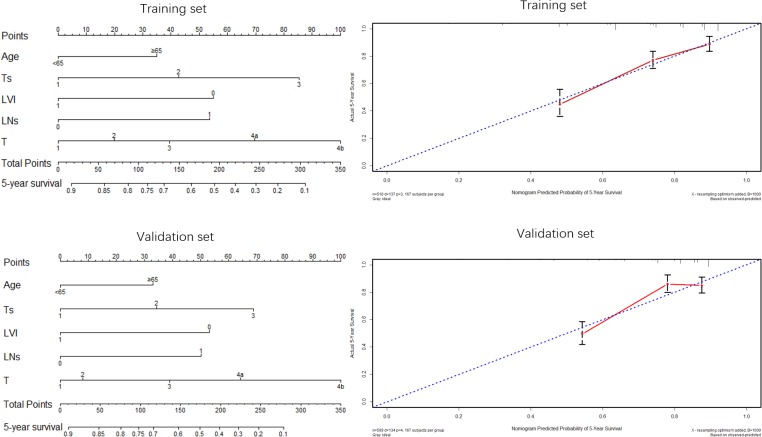
Nomogram plots and calibration curves based on age, Ts, LVI, LNs and T stage

**Figure 6:**
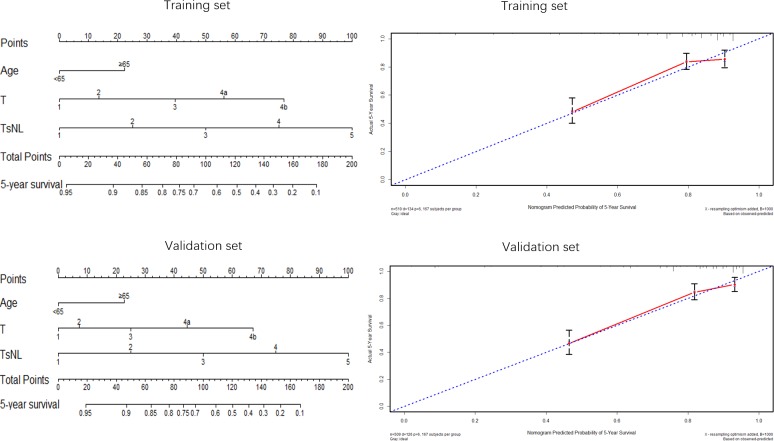
Nomogram plots and calibration curves based on the TsNL staging system

Nomogram was applied to predict 5-year OS of patients (Figures 5 & 6). Both in the training set and validation set, factors such as age, Ts, LVI, LNs and T stage, were enrolled in the nomogram plots (Figure 5), demonstrating that these five factors were independent factors and that age ≥65, larger tumor size, positive LVI and LNs < 14 as well as advanced T stage were adverse prognostic factors, which was consistent with the aforementioned results displayed by Cox regression analyses in this study. Nomograms based on TsNL staging system for the training set and validation set were illustrated in Figure 6, and the corresponding calibration curves in the two sets suggested that the predictive probability of 5-year survival were much more closely to the actual 5-year survival than that of calibration curves produced in Figure 5.

Moreover, the concordance index (C-index) in R was used to compare the prognostic accuracy between TsNL stage and T stage system. To be specific, TsNL staging system (c-index = 0.834, 95%CI: 0.790-0.881, Figure 4) was found to be significantly superior to T stage in the training set (c-index = 0.793, 95%CI: 0.723-0.827, Figure 2) in survival prediction accuracy (*p* < 0.05), and similar result also appeared in the validation set as shown in Figure 2 &4.

## DISCUSSION

For lymph node-negative GC patients who underwent curative gastrectomy, T stage has been considered as the most important prognostic predictor according to the TNM staging system [2, 3]. In this study, in addition to T stage, clinicopathologic features such as age, tumor size (Ts), lymphovascular invasion (LVI) and lymph nodes count (LNs), were identified as independent prognostic factors by multivariate Cox regression analysis.

The optimal cutoff points for Ts were 3cm and 5cm in this study, which could produce minimum p value by log-rank and maximum discrimination ability on prognostic prediction both in the training set and validation set. As an important prognostic factor, Ts has already been integrated into the TNM staging system for liver cancer, lung cancer and breast cancer, but not for gastric cancer. In our previous study, Ts was found no superiorities than T stage for node-negative GC patients, but it was more accurate in combination with N stage than TNM staging system in survival prediction [11]. Moreover, the status of lymphovascular invasion (LVI) has been previously observed as an important factor, influencing the clinical outcome of gastric cancer patients who underwent radical gastrectomy, and the presence of LVI has been identified to be of significantly relevance to a poor overall survival for advancer GC patients in several studies [17–19], while some researchers proposed that LVI was just associated with the survival prognosis for early GC patients or node-negative GC patients [6–9]. Our findings revealed a significant difference between node-negative GC patients with LVI and those without LVI on overall survival, which was in accordance with the latter point of view.

The removal of no less than 15 regional lymph nodes count (LNs) at the time of lymphadenectomy for gastric cancer during surgical treatment has been largely demonstrated to improve survival outcomes [12, 14, 20–22]. Given that GC patients might be staged incorrectly because of an insufficient number of LNs, which could lead to miss an inappropriate adjuvant therapy [23], a minimum of 15 LNs is recommended to be retrieved in lymphadenectomy for the sake of nodal metastatic status determination for GC patients in the NCCN guidelines and JGCA [2, 3, 24]. For node-negative GC patients, the number of LNs was also found to be significantly associated with the prognosis, but there have long been controversies over how many LNs should be removed in radical gastrectomy, with the cutoff numbers ranging from 15 to 25 in several studies [12, 21, 25, 26]. Theoretically, an increasing number of LNs indicates a comparatively accurate N stage, especially for lymph node-negative GC patients, due to that these patients have a great risk of being misclassified when few nodes are harvested and their clinical survival outcomes are likely to be changed if they are given timely adjuvant therapy because of the stage migration from negative to positive lymph nodes. In our study, the LNs was demonstrated to be an independent prognostic factor as well, but the optimal cutoff point was 14, which was inconsistent with previous studies. This might be explained by that for the total number of LNs retrieved in lymph-node negative GC patients were less than that in node-positive patients. Studies on population registries have reported that only18-31% of cases were harvested 15 or more LNs [26, 27]. That could be also the reason why node-negative (N0) stage is defined as any gastric cancer with all examined LNs negative, regardless of the total number of LNs in the 7^th^ edition of the TNM classification [2].

Furthermore, T stage and Ts were both demonstrated to be independent prognostic factors in our study and showed similar prognostic power independently. However, logistic regression analyses were performed in this study to identify the risk factors for T stage, Ts, LNs and LVI as well, indicating that T stage was closely correlated to Ts and that multicollinearity between them was found, which reminded us that T and Ts could not be integrated into one staging model. That was one of the reason why T stage was not taken into account for our TsNL staging system. In order to make the utmost use of these independent factors to offer detailed prognostic information, we integrated the independent factors, Ts, LNs and LVI, together to propose a new staging system, TsNL, which could provide powerful survival discrimination ability and enhance the prognostic accuracy for node-negative GC patients. The patients in this study were divided into five stages according to the TsNL staging system both in the training set and validation set, and TsNL stage was significantly associated with clinicopathologic features, such as gender, age, tumor location, macroscopic type, tumor differentiation and perineural invasion as well as T stage. Patients with late TsNL stage were likely to be diagnosed with worse biological behavior and more aggressive features than those with early TsNL stage.

Nomogram, as an effective method to evaluate survival prognosis for patients, was used in this study to show visually the prognostic significance of some important factors on the GC patients. As independent prognostic factors, age, Ts, LVI, LNs and T stage, were enrolled in the nomogram plots. Nomograms and calibration curves based on TsNL staging system revealed a much closer predictive probability of 5-year survival to the actual 5-year survival, according to which we could believe that nomogram based on TsNL staging system showed an improved predictive capability of 5-year overall survival. Additionally, the prognostic accuracy between TsNL stage and T stage system was compared using C-index, as the T stage was the most important prognostic predictor for node-negative GC patients according to the TNM staging system. C-indexes for TsNL stage were observed significantly larger than that for T stage both in the training set and validation set in our study, which illustrated that TsNL stage was more accurate in prognostic prediction than T stage. Given that selection of an appropriate therapy strategy for GC patients in accordance with tumor stage is extremely important and essential to optimize patient prognosis, perhaps node-negative GC patients could benefit a lot from this new staging system, not only because of its powerful discrimination ability in survival estimation but also due to its improved accuracy in prognostic prediction.

There were also limitations in our study. First of all, our findings we got were just on the basis of a retrospective single-center study, which could have been observed by chance in spite of the large sample. In addition, we were lack of another separated validation set to evaluate the predictive power of TsNL staging system. Therefore, large scale and prospective multicenter studies are needed to evaluate the TsNL staging system can whether or not be an important prognostic index for the node-negative GC patients before stronger statement can be done.

In conclusion, age, T stage, Ts, LNs and LVI in our study were independent prognostic factors for lymph node-negative GC patients. Moreover, composed of Ts, LNs and LVI, the TsNL staging system, which was closely associated with clinicopathologic features, could improve the prognostic prediction accuracy in node-negative GC patients.

## PATIENTS AND METHODS

### Patients

The West China Hospital Research Ethics Committee approved the retrospective analysis of anonymous data involved in this study. The data retrieval of this study was based on the Surgical Gastric Cancer Patient Registry in West China Hospital [28]. Patient records were anonymized and de-identified prior to analysis, and signed patient informed consent was waived per the committee approval because of the retrospective nature of the analysis.

**Figure 7:**
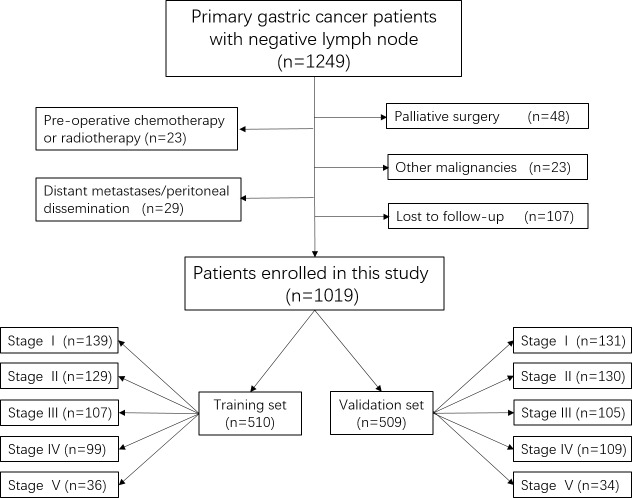
The flow chart of patients in this study

From 2000 January to 2010 December, a total of 1249 consecutive lymph node-negative GC patients who received gastrectomy at the Department of Gastrointestinal Surgery, West China Hospital, were retrospectively evaluated in this study. The diagnosis of primary gastric cancer for all patients was confirmed by upper gastrointestinal endoscopy and biopsy. Patients were excluded on the condition that: (1) patients who underwent palliative surgery with positive residual margins; (2) patients with any pre-operative chemotherapy or radiotherapy; (3) patients with another malignancy or any other life-threatening diseases diagnosed during three years prior to the operation; (4) patients with surgical findings of distant metastasis or peritoneal dissemination. (5) patients who were lost to follow-up. Finally, 109 patients were lost to follow-up and the follow-up rate was 91.43% in this study. A total of 1019 patients were enrolled in this study as shown in Figure 7. The clinicopathological characteristics including of gender, age, tumor location, macroscopic type, tumor differentiation, perineural invasion, T stage, defined as the depth of tumor invasion according to the Japanese gastric cancer treatment guidelines 2010 (version 3) [3], and follow-up information were collected.

### Definition of TsNL staging system

Tumor size (Ts), was divided into three groups (Ts1: ≤3cm, Ts2: 3-5cm, Ts3: ≥5cm) by the cutoff points of 3.0cm and 5.0cm using X-tile, and the lymph nodes count (LNs) was categorized into N0 (LNs≥14) and N1 (LNs < 14) by the cutoff point of 14. Lymphovascular invasion (LVI) was defined as status of tumor invasion of lymphatics or small veins, and L0 was regarded as negative LVI whereas L1 symbolized positive LVI. Consequently, TsNL staging system shown in Table 3, was designed as combination of Ts, LNs and LVI, based on which patients were randomly categorized into the training set and the validation set by the sample size ratio 1:1 using X-tile.

### Statistical analysis

Optimal cutoff points for survival were determined by minimum P value from log-rank χ^2^ statistics using the X-tile program (Version 3.1.2, Yale University) [29]. Chi-square test in the SPSS version 19.0 was applied to analyze unordered categorical variables, whereas Mann-Whitney U test was performed to evaluate ranked variables. Logistic regression analysis was used to analyze the multicollinearity or multivariate correlation. Univariate and multivariate survival analyses were performed by Cox's proportional hazard regression model with conditional backward stepwise. The cumulative survival rates were calculated using the Kaplan-Meier method and life-table in the SPSS, with subgroups compared by the log-rank test through GraphPad Prism 5. Nomogram and calibration curve were displayed with the package of Regression Modeling Strategies *(URL* *http://CRAN.R-project.org/package* *= rms)* in R (version3.1.2.*URL* *http://www.R-project.org/*.) Comparisons between the different staging systems for the prognostic prediction were conducted with the package of Harrell Miscellanceous *(URL* *http://CRAN.R-project.org/package* *= Hmisc.)* and were evaluated by the concordance index (C-index). The larger the C-index, the more accurate was the prognostic prediction [30]. A *p* value of < 0.05 (two side) was defined to be statistically significant.
